# Supplementary material for: Identification of photoperiod-induced specific miRNAs in the adrenal glands of Sunite sheep (Ovis aries)
Source: Front Vet Sci. 2022 Jul 22;9:888207. doi: 10.3389/fvets.2022.888207 (PMC9354845; doi:10.3389/fvets.2022.888207)
Supplement: Supplementary file 1 [file Data_Sheet_1.ZIP › Supplementary file/miRDeep2 file.zip!/miRDeep2 file/Know_Structure/LP42A3/oar-mir-411a.pdf]

miRBase precursor : oar-mir-411a
Total read count : 61306
oar-miR-411a-5p read count : 56265
oar-miR-411a-3p read count : 5031
remaining reads : 10

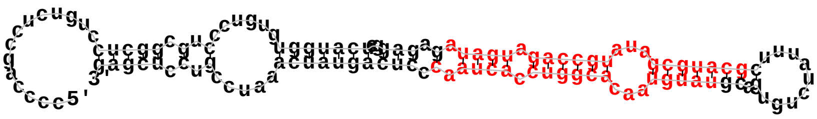

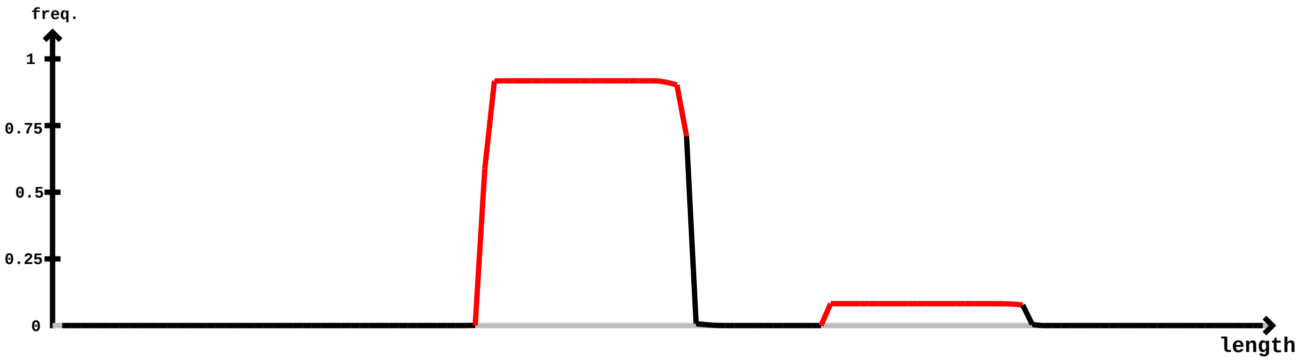


```
                                                                                     oar-miR-411a-3p
                                          oar-miR-411a-5p
5'- ccccagccucuguccucggcgucccugugugguacucggagagauaguagaccguauagcguacgcuuuaucugugacguauguaacacgguccacuaacccucaguaucaaauccguccucgag   -3'   exp
    .............(((((.(.(......(((((((..(((.(.((((.((((((...((((((((.........).)))))))...)))))).)))).).))))))))))).....).).)))))   reads   mm
    ......................cccugugugguacucggagagau................................................................................   2       0
    .......................ccugugugguacucggagaga.................................................................................   2       0
    .......................ccugugugguacucggagagau................................................................................   1       0
    ........................cugugugguacucggag....................................................................................   1       0
    ........................cugugugguacucggagaga.................................................................................   1       0
    ........................cugugugguacucggagagau................................................................................   1       0
    .........................................agauaguagaccguauagcgu...............................................................   5       0
    .........................................agauaguagaccguauagcgua..............................................................   1       0
    .........................................agauaguagaccguauagcguacg............................................................   1       0
    ..........................................gauaguagaccguauagcgu...............................................................   4       0
    ..........................................gauaguagaccguauagcgua..............................................................   2       0
    ..........................................gauaguagaccguauagcguac.............................................................   12      0
    ..........................................gauaguagaccguauagcguacg............................................................   15      0
    ...........................................auaguagaccguauagc.................................................................   2       0
    ...........................................auaguagaccguauagcgu...............................................................   324     0
    ...........................................auaguagaccguauagcgua..............................................................   414     0
    ...........................................auaguagaccguauagcguac.............................................................   7334    0
    ...........................................auaguagaccguauagcguacg............................................................   27772   0
    ...........................................auaguagaccguauagcguacgc...........................................................   98      0
    ...........................................auaguagaccguauagcguacgcu..........................................................   50      0
    ...........................................auaguagaccguauagcguacgcuuu........................................................   1       0
    ............................................uaguagaccguauagcgu...............................................................   32      0
    ............................................uaguagaccguauagcgua..............................................................   163     0
    ............................................uaguagaccguauagcguac.............................................................   4426    0
    ............................................uaguagaccguauagcguacg............................................................   15302   0
    ............................................uaguagaccguauagcguacgc...........................................................   127     0
    ............................................uaguagaccguauagcguacgcu..........................................................   114     0
    ............................................uaguagaccguauagcguacgcuu.........................................................   29      0
    ............................................uaguagaccguauagcguacgcuuu........................................................   6       0
    .............................................aguagaccguauagcgua..............................................................   1       0
    .............................................aguagaccguauagcguac.............................................................   4       0
    .............................................aguagaccguauagcguacg............................................................   18      0
    .............................................aguagaccguauagcguacgcu..........................................................   4       0
    .............................................aguagaccguauagcguacgcuu.........................................................   1       0
```

oar-miR-411a-3p

oar-miR-411a-5p

ccccagccucuguccucggcgucccugugugguacucggagagauaguagaccguauagcguacgcuuuaucugugacguauguaacacgguccacuaacccucaguaucaaauccguccucgag

....aguagaccguauagcguacgcuuu.. 1 0
....guagaccguauagcguacg.. 2 0
....cuuuaucugugacguauguaac...................................... 1 0
....ugacguauguaacacgguccacu............................ 1 0
....cguauguaacacgguccacua........................... 1 0
....cguauguaacacgguccacuaa.......................... 2 0
....guauguaacacgguccacuaac......................... 3 0
....uauguaacacgguccacu............................ 27 0
....uauguaacacgguccacua........................... 58 0
....uauguaacacgguccacuaa.......................... 214 0
....uauguaacacgguccacuaac......................... 4482 0
....uauguaacacgguccacuaacc........................ 242 0
....auguaacacgguccacuaac......................... 2 0
